# Effect of Ultraviolet-Ozone Treatment on MoS_2_ Monolayers: Comparison of Chemical-Vapor-Deposited Polycrystalline Thin Films and Mechanically Exfoliated Single Crystal Flakes

**DOI:** 10.1186/s11671-019-3119-3

**Published:** 2019-08-15

**Authors:** Changki Jung, Hae In Yang, Woong Choi

**Affiliations:** School of Materials Science & Engineering, Kookmin University, Seoul, 02707 South Korea

**Keywords:** MoS_2_, Monolayer, Single crystal, Polycrystalline, UV-O_3_ treatment

## Abstract

We report the different oxidation behavior between polycrystalline chemical-vapor-deposited and mechanically exfoliated single crystal MoS_2_ monolayers by ultraviolet-ozone treatment. As ultraviolet-ozone treatment time increased from 0 to 5 min, photoluminescence emission and Raman modes of both MoS_2_ disappeared, suggesting structural degradation by oxidation. Analysis with optical absorbance and X-ray photoelectron spectroscopy suggested the formation of MoO_3_ in both MoS_2_ after ultraviolet-ozone treatment. In addition, ultraviolet-ozone treatment possibly led to the formation of oxygen vacancies, molybdenum oxysulfide, or molybdenum sulfates in chemical-vapor-deposited MoS_2_. The measurement of electrical resistance after ultraviolet-ozone treatment suggested the transformation of chemical-vapor-deposited MoS_2_ into doped MoO_3_ and of mechanically exfoliated MoS_2_ into negligibly doped MoO_3_. These results demonstrate that the crystallinity of monolayer MoS_2_ can strongly influence the effect of ultraviolet-ozone treatment, providing important implications on the device integration of MoS_2_ and other two-dimensional semiconductors.

**Electronic supplementary material:**

The online version of this article (10.1186/s11671-019-3119-3) contains supplementary material, which is available to authorized users.

## Introduction

There is a great interest in transition metal dichalcogenides (TMDs), such as MoS_2_, since they offer an attractive possibility for various device applications including transistors, optoelectronic devices, heterojunction structures, sensors, and electrocatalysis [1, 2]. The existence of direct bandgaps in monolayer TMDs makes these two-dimensional semiconductors especially promising for optoelectronic devices [3, 4]. However, critical challenges to fabricate TMD-based optoelectronic devices such as phototransistors include the deposition of high-*k* dielectrics on TMDs and the doping of TMDs. Because of the absence of dangling bonds on the surface of TMDs, it is challenging to deposit high-*k* dielectrics on TMDs [5]. Moreover, the doping of TMDs is also challenging as the substitutional doping used for bulk semiconductors such as silicon modifies the two-dimensional structure and properties of monolayer TMDs [6].

To overcome these difficulties, surface functionalization of TMDs by O_2_ plasma [7, 8] or ultraviolet-ozone (UV-O_3_) [9–11] has been suggested. While these methods can functionalize the surface of MoS_2_ by surface oxidation, they can simultaneously influence the structure and properties of monolayer MoS_2_ [12–16]. For example, oxidation by O_2_ plasma or UV-O_3_ treatment altered the Raman vibration modes and photoluminescence (PL) emission of monolayer MoS_2_ [12, 16]. However, as most studies were based on micrometer-scale monolayer MoS_2_ flakes obtained by mechanical exfoliation from bulk single crystals, little has been known on their interaction with large-area monolayer MoS_2_ thin films, which are typically polycrystalline. Grain boundaries in polycrystalline monolayer MoS_2_ may allow higher reactivity with UV-O_3_ than that of single crystal, resulting in different oxidation behavior. Therefore, in this study, we explore the effect of UV-O_3_ treatment on MoS_2_ monolayers by directly comparing the oxidation behavior of polycrystalline chemical vapor deposition (CVD) thin films and mechanically exfoliated single crystal flakes. We systematically investigate the PL and Raman spectra of both MoS_2_ monolayers for different duration of UV-O_3_ exposure. We also investigate the oxidation behavior of both MoS_2_ monolayers during UV-O_3_ treatment with X-ray photoelectron spectroscopy (XPS). We further measure electrical resistance of pristine and UV-O_3_-treated MoS_2_ monolayers to understand the effect of UV-O_3_ treatment on MoS_2_ monolayers.

## Methods

**Fig. 1:**
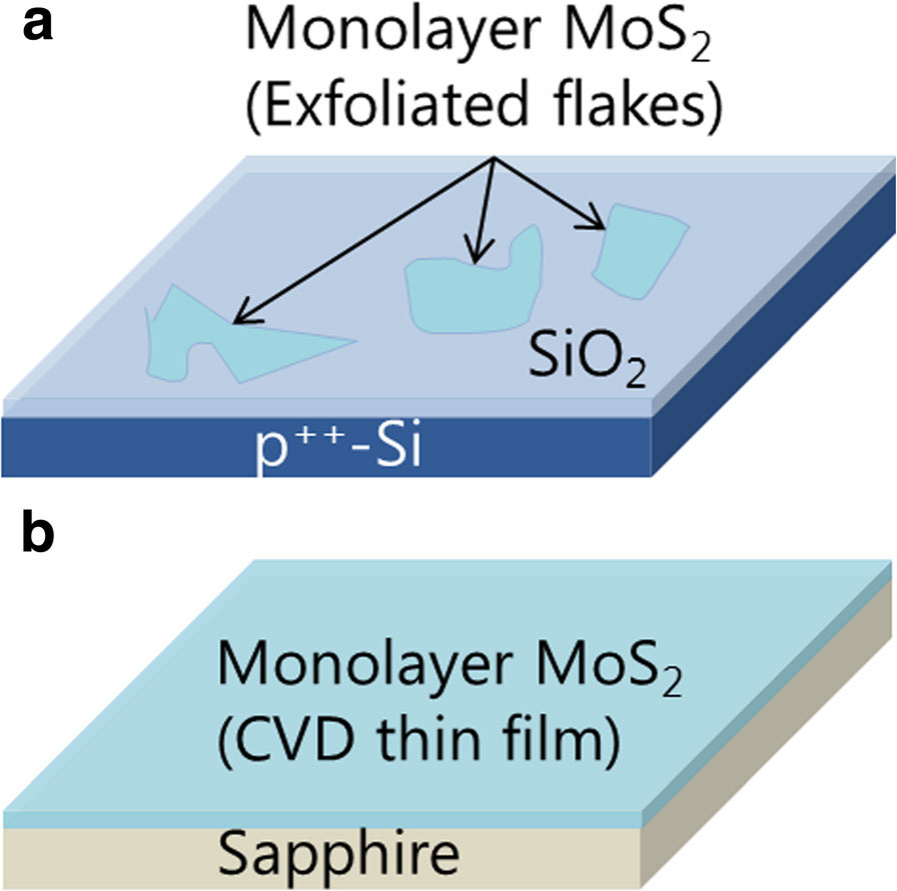
Schematic structures of MoS_2_ monolayers: **a** mechanically exfoliated flakes on SiO_2_/Si substrates and **b** CVD thin films on sapphire substrates

Monolayer MoS_2_ thin films were deposited on (0001)-oriented sapphire substrates (~ 1.5 × 1 cm^2^) by CVD in a two-zone tube furnace. MoO_3_ (99.98%, Sigma-Aldrich) and S (99.98%, Sigma-Aldrich) powders in two separate Al_2_O_3_ boats were used as precursors. MoO_3_ powder (14 mg) was placed upstream at zone 1 (750 °C) and S powder (1.4 g) was placed at the upstream entry of the furnace. Substrates were placed downstream at zone 2 (700 °C). MoO_3_ powder was heated at a rate of 15 °C min^−1^ and substrates were heated at 38 °C min^−1^. After 30-min deposition, the furnace was slowly cooled down to room temperature. Ar flow of 100 sccm and a pressure of ~ 0.5 Torr were maintained during deposition. Monolayer MoS_2_ flakes were obtained by the gold-mediated exfoliation method [17] from bulk MoS_2_ crystals (2D Semiconductors) and transferred on highly-doped Si substrates with thermally grown SiO_2_ (300 nm). Figure 1 shows schematic structures of both MoS_2_ monolayers on substrates. The thickness of monolayer MoS_2_ was measured using atomic force microscopy (AFM, Park Systems XE-100). The crystallinity of bulk MoS_2_ crystals and CVD MoS_2_ thin films was investigated by X-ray diffraction (XRD, Bruker D8 Discover with Cu-Kα radiation) and transmission electron microscopy (TEM, FEI Titan 80–300 at 300 kV), respectively.

MoS_2_ monolayers were exposed to UV-O_3_ (SEN LIGHTS PL16–110, 185 nm and 254 nm) for 0–5 min at the irradiance of 58 mW cm^−2^. Optical absorbance was measured by UV-visible spectroscopy (PerkinElmer Lambda 35). Raman/PL spectroscopy (Horiba Jobin-Yvon LabRam Aramis) were measured on pristine and UV-O_3_-treated MoS_2_ monolayers with a 532-nm laser and a beam power of 0.5 mW. XPS (Thermo Scientific K-Alpha) was carried out using a monochromatic Al K_α_ x-ray source (*hν* = 1486.7 eV) with a take-off angle of 45°, a pass energy of 40 eV, and a spot size of 400 μm in diameter. For all samples, C 1s and O 1s were observed presumably because they are exposed to atmosphere before loaded to ultrahigh vacuum chamber for XPS analysis. Adventitious carbon (C 1s at 284.8 eV) was used as a charge correction reference for XPS spectra. The energy resolution is 0.7 eV measured using the full width at half-maximum intensity of the Ag 3d_5/2_ peak. MoS_2_ samples were exposed to atmosphere while they were brought to XPS equipment. Although in situ XPS analysis could provide more accurate information, it was unavailable in this work. For peak deconvolution and background subtraction, Thermo Scientific Avantage Data System software was used. Gaussian functions were used to fit XPS spectra.

To measure the electrical resistance of MoS_2_ monolayers, Au contacts (100 × 100 μm^2^, 70 nm thick) were deposited on top of MoS_2_ by electron-beam evaporation. Spin-coated photoresist on top of Au layer was then patterned by conventional photolithography to form opening areas for subsequent etching. After Au in opening areas was removed by wet etching in aqua regia, remaining photoresist was removed in acetone. Then, the devices were annealed at 200 °C for 2 h in a tube furnace (100 sccm Ar and 10 sccm H_2_) to remove photoresist residue and to decrease contact resistance. Electrical resistance was calculated with current-voltage (*I*–*V*) measurement (Keithley 4200-SCS) in atmospheric environments.

## Results and Discussion

Beside AFM measurement, PL and Raman spectra are measured to confirm the formation of MoS_2_ monolayers. Because of its direct bandgap, MoS_2_ monolayers allow PL emission at ~ 1.88 eV [3, 4]. In addition, the frequency difference between the two characteristic Raman A_1g_ and E^1^_2g_ modes of MoS_2_ monolayers is less than 20 cm^−1^ [18]. In Fig. 3, the PL emission of pristine MoS_2_ at ~ 1.88 eV indicates that both MoS_2_ are monolayers. In Fig. 4, pristine MoS_2_ exhibits the frequency difference between 19.6 and 19.9 cm^−1^ implying monolayer MoS_2_. XRD and TEM analysis indicated the single crystal nature of bulk MoS_2_ crystals and polycrystalline nature of our monolayer MoS_2_ thin films (Additional file 1: Figure S1). The grain size of monolayer MoS_2_ thin films is ~ 10 nm [19].

**Fig. 2:**
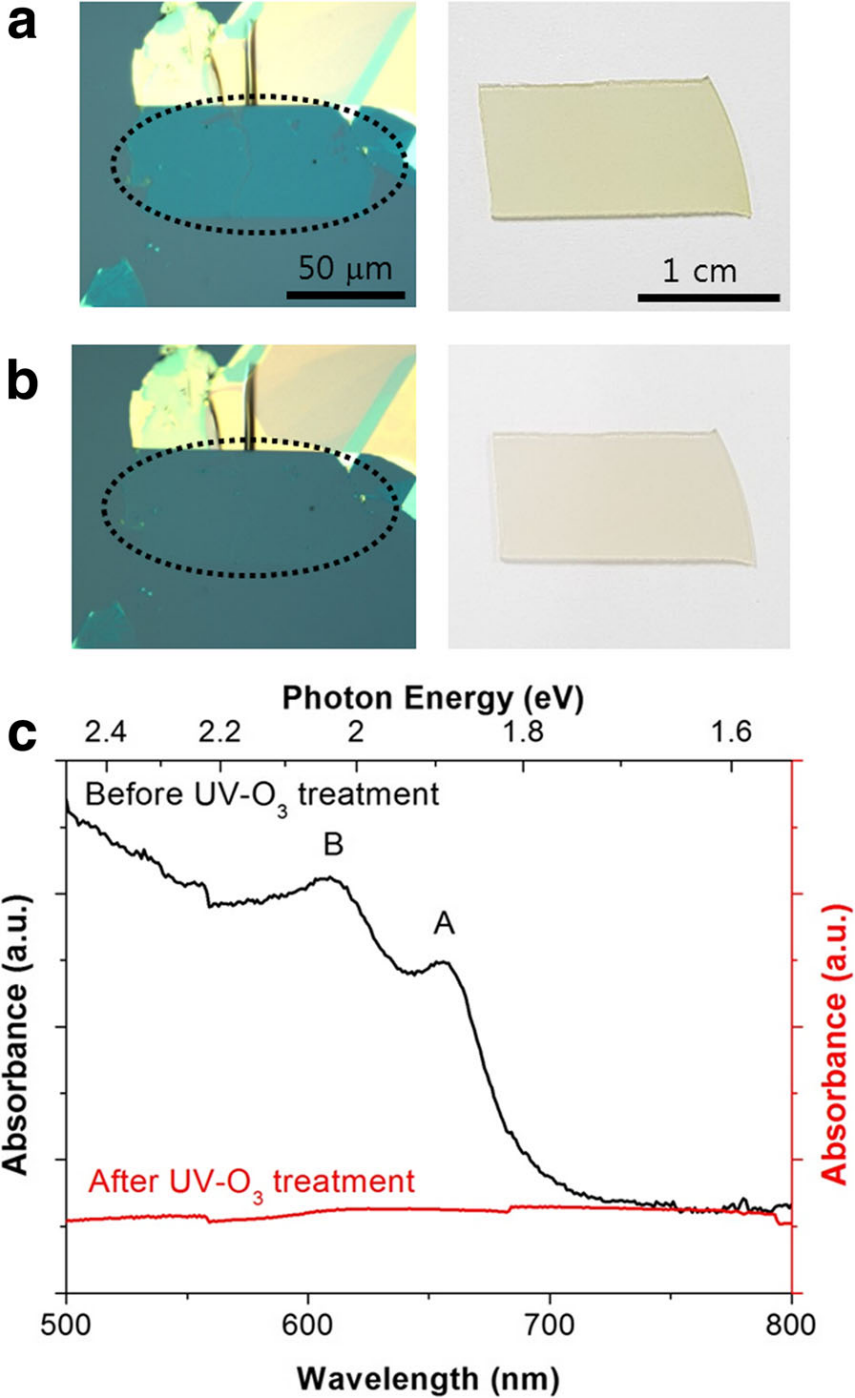
Mechanically exfoliated flakes and CVD thin films of MoS_2_ monolayers **a** before and **b** after 5-min UV-O_3_ treatment (dotted region indicates monolayer), **c** optical absorbance of CVD thin films before and after 5-min UV-O_3_ treatment

After UV-O_3_ treatment, MoS_2_ monolayers change their color and become transparent. In Fig. 2a, b, both exfoliated flakes and CVD thin films become transparent after 5-min UV-O_3_ treatment. The absorbance spectrum of MoS_2_ thin films in Fig. 2c clearly shows the difference after UV-O_3_ treatment. (The absorbance of exfoliated MoS_2_ flakes could not be measured with UV-visible spectroscopy as the size of flakes was too small.) While pristine MoS_2_ thin films show absorbance peaks due to excitonic transitions (A and B) [3, 4], 5-min UV-O_3_-treated MoS_2_ thin films do not exhibit any absorbance peaks at all for the same range of wavelength. Because lightly yellow-green MoS_2_ thin films become transparent to visible light after 5-min UV-O_3_ treatment, we expect the energy bandgap of pristine monolayer MoS_2_ (~ 1.88 eV) to become wider after UV-O_3_ treatment (> ~ 3 eV). As this is in good agreement with the wide bandgap of MoO_3_ (> 2.7 eV) [20], the transparent UV-O_3_-treated MoS_2_ suggests the formation of MoO_3_ after 5-min UV-O_3_ treatment.

**Fig. 3:**
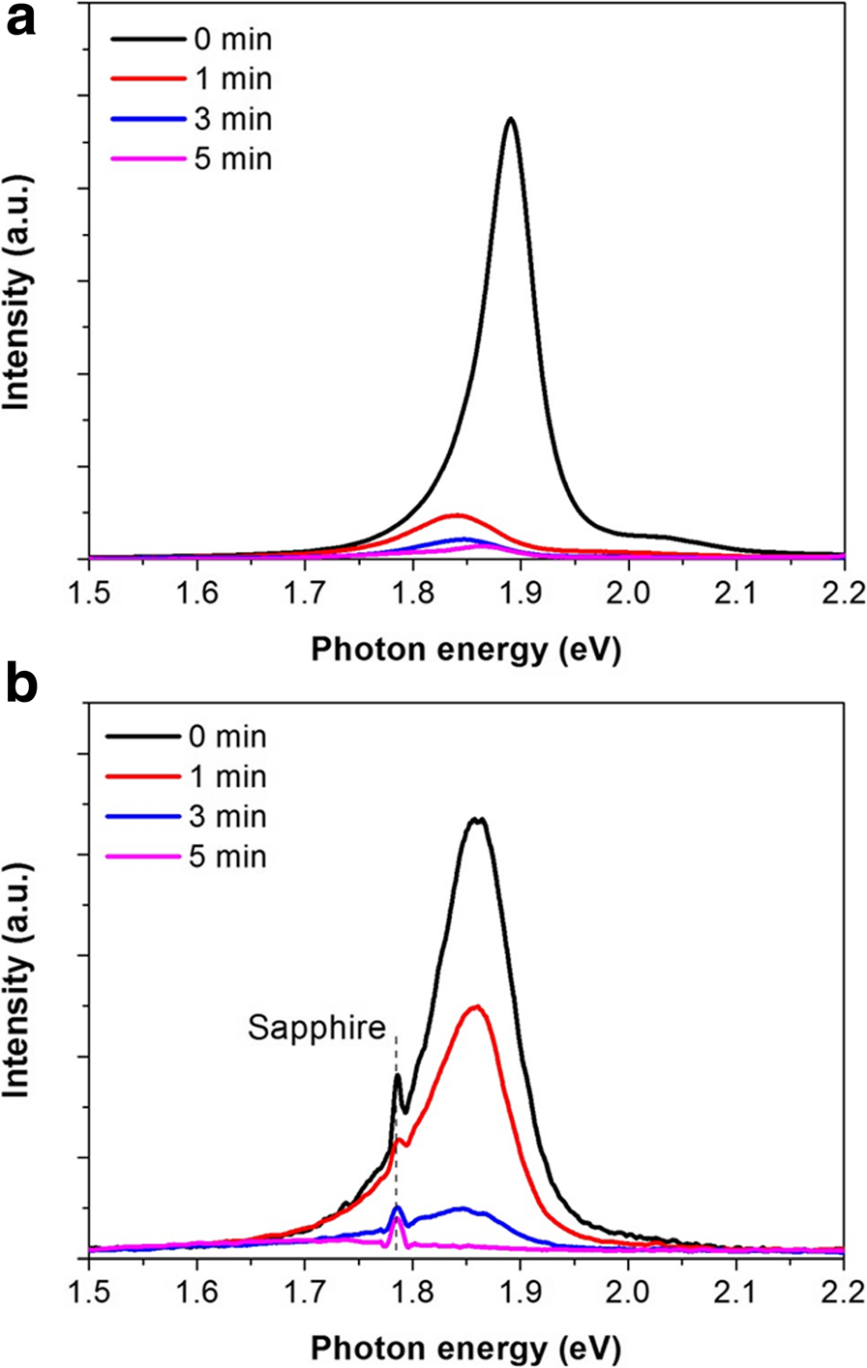
PL spectra of MoS_2_ monolayers **a** mechanically exfoliated flakes on SiO_2_/Si substrates and **b** CVD thin films on sapphire substrates after UV-O_3_ treatment for 0, 1, 3, and 5 min

We then investigate the effect of UV-O_3_ treatment on the PL emission of MoS_2_ monolayers. Figure 3 shows the PL spectra of CVD MoS_2_ thin films and exfoliated MoS_2_ single crystal flakes after UV-O_3_ exposure for 0, 1, 3, and 5 min, respectively. The intensity of PL emission decreases significantly with UV-O_3_ treatment time and eventually PL is fully quenched for the 5-min-treated MoS_2_ monolayers. These results suggest the formation of oxides or defects allowing non-radiative recombination after UV-O_3_ treatment. As MoS_2_ monolayers become transparent after UV-O_3_ treatment, the formation of wide bandgap semiconductor MoO_3_ is reasonably expected. The energy of PL emission of pristine MoS_2_ is 1.88 eV for exfoliated flakes and 1.86 eV for CVD films. This slight difference is probably due to the effect of underlying substrates as substrates can strongly influence the Raman and PL emission [21]. The wider width of PL emission peak in CVD monolayers suggests higher defect density. Interestingly, further negative shift of PL emission peak is observed in single crystal MoS_2_ flakes (~ 50 meV) than in CVD thin films (by ~ 10 meV) after UV-O_3_ treatment. As the negative shift of PL emission is comparable with trion binding energy (10–40 meV) of MoS_2_ [22], this may be due to different concentrations of trion (neutral excitons accepting an electron or a hole) formed by oxidation-induced doping [23, 24]. (In this work, single crystal MoS_2_ flake is more conductive than CVD MoS_2_, suggesting higher doping levels in single crystal MoS_2_.) The higher doping level in single crystal MoS_2_ flakes will allow high concentration of trions, of which recombination will dominate their PL emission. In contrast, the lower doping level in CVD MoS_2_ thin films will allow low concentration of trions. Hence, their PL emission will be dominated by the recombination of neutral excitons. However, as the negative shift of PL emission may also be related to the effect of underlying substrates or strains, more systematic investigation is needed in the future.

**Fig. 4:**
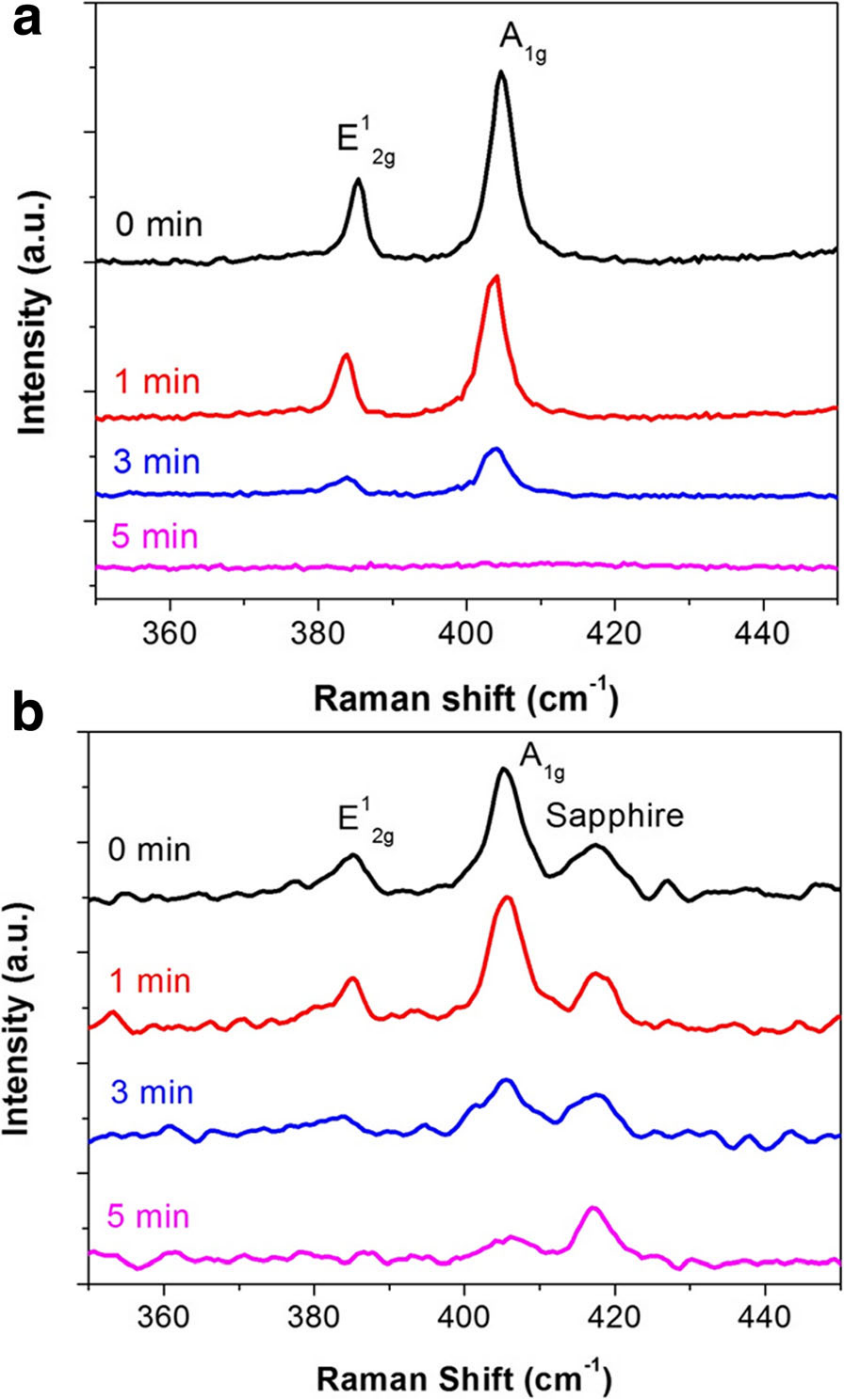
Raman spectra of MoS_2_ monolayers **a** mechanically exfoliated flakes on SiO_2_/Si substrates and **b** CVD thin films on sapphire substrates after UV-O_3_ treatment for 0, 1, 3, and 5 min

Next, to investigate the structural degradation by UV-O_3_ treatment, we measure the Raman spectra of MoS_2_ monolayers after UV-O_3_ treatment for 0, 1, 3, and 5 min, respectively (Fig. 4). The intensity of both E^1^_2g_ and A_1g_ modes decreases as the treatment time increases. While the frequency difference between E^1^_2g_ and A_1g_ modes remains unchanged for 0–5 min of UV-O_3_ treatment time, the two Raman modes almost completely disappear after 5-min treatment, suggesting severe structural distortion and degradation. AFM analysis indicates an increase of surface roughness after UV-O_3_ treatment (Additional file 1: Figure S2), which is consistent with the oxidation of MoS_2_ [23].

**Fig. 5:**
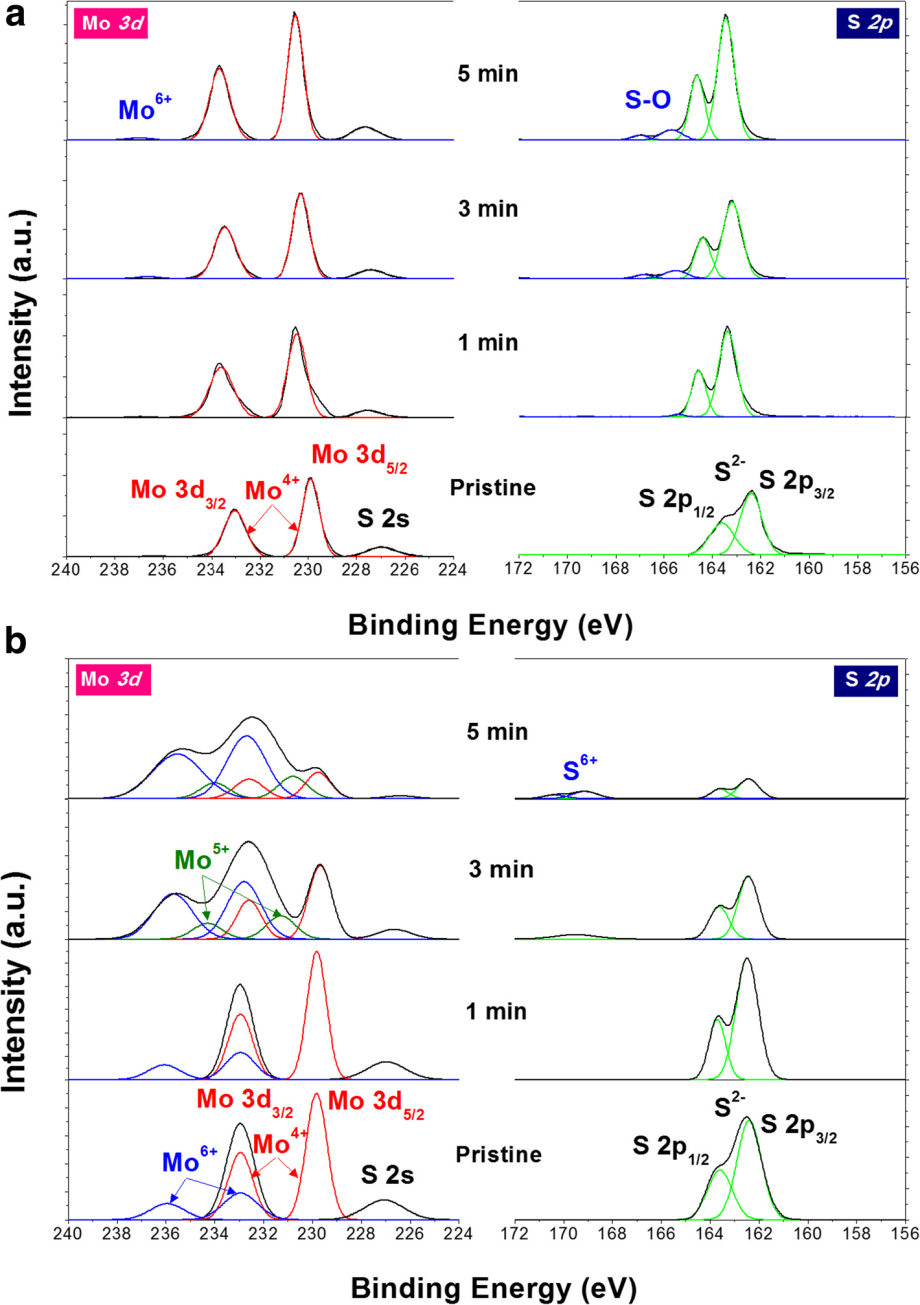
XPS spectra of MoS_2_ **a** bulk single crystal and **b** CVD thin films on sapphire substrates after UV-O_3_ treatment for 0, 1, 3, and 5 min

To further investigate the structural degradation of MoS_2_ monolayers by UV-O_3_ treatment, we measure XPS spectra of MoS_2_. Because the beam size of XPS is much larger than the size of single-layer MoS_2_ flakes, XPS spectra for single crystal MoS_2_ flakes are obtained from large-area MoS_2_ single crystals (~ 1 cm in size and ~ 100 μm in thickness). Figure 5 shows the XPS spectra in Mo 3d and S 2p regions for bulk single crystal and CVD MoS_2_ thin films, respectively. The existence of Mo^4+^-state of pristine MoS_2_ can be observed from the binding energy of Mo 3d_3/2_ and Mo 3d_5/2_ orbitals. After UV-O_3_ exposure, the intensity of Mo^6+^-state at 235.9 eV further increases with UV-O_3_ treatment time indicating the expanded formation of Mo-O bonding and MoO_3_. There are four distinct differences between Fig. 5a and b in Mo 3d region. (1) In Fig. 5b, Mo^6+^-state at 235.9 eV in pristine MoS_2_ thin films is probably due to residual oxide formed during or after CVD process. (2) The intensity of Mo^4+^ and S 2 s peaks decrease in CVD MoS_2_ thin films with longer UV-O_3_ exposure. However, the intensity of Mo^4+^ and S 2 s peaks does not change with UV-O_3_ treatment time in large MoS_2_ single crystals as XPS can still detect Mo^4+^ and S 2 s peaks from MoS_2_ underneath the oxidized top surface. (3) In single crystal MoS_2_, the binding energy of Mo^4+^-state shows further positive shift than that in CVD MoS_2_ thin films suggesting higher n-type doping [25]. The peak shift after the oxidation of MoS_2_ in this work (0.41–1.09 eV) is comparable to that in literature (0.6–1.1 eV) [23, 24]. (To prevent charging effect, which may induce similar positive shift, we used a flood gun during XPS measurement.) (4) In CVD MoS_2_ thin films, the peaks of Mo^5+^-state also appear with UV-O_3_ treatment suggesting possibly the formation of oxygen vacancies [26] or molybdenum oxysulfide MoO_x_S_y_ [27]. These results can be understood by the oxidation of Mo^4+^-state in MoS_2_ into higher oxidation states (Mo^5+^ and Mo^6+^) with UV-O_3_ exposure. This is also consistent with the XPS results on polycrystalline multilayer MoS_2_ thin films after O_2_ plasma or UV-O_3_ treatment [26, 28, 29].

In S 2p region, the existence of S^2−^-state can be observed from the binding energy of S 2p_1/2_ and S 2p_3/2_ orbitals in pristine MoS_2_. The binding energy of S^2−^-state in single crystal MoS_2_ shows further positive shift than that in CVD MoS_2_ thin films suggesting higher n-type doping [25]. Although S-O bond is observed at ~ 165 eV in UV-O_3_-treated single crystal MoS_2_, it is below the detection limit in CVD thin films. Instead, a new doublet peak of sulfur oxidation state appears at higher binding energy (~ 169 eV) in CVD thin films after UV-O_3_ treatment for 3 min. This new doublet corresponds to the S 2p peaks of oxidized sulfur S^6+^, suggesting possibly the formation of various molybdenum sulfates Mo (SO_4_)_x_ [28]. While the intensity of S^2−^ doublet keeps decreasing with longer UV-O_3_ exposure, the intensity of S^6+^ doublet further increases after 5-min UV-O_3_ treatment, suggesting further conversion of S^2−^ into higher oxidation state (S^6+^) by oxidation. Similarly with Mo^4+^ peaks, the intensity of S^2−^ peaks does not change with UV-O_3_ treatment time in large MoS_2_ single crystals. The existence of S^6+^-state after O_2_ plasma or UV-O_3_ treatment is inconsistent in literature. Its existence was reported in polycrystalline multilayer MoS_2_ thin films after O_2_ plasma treatment [28]. However, it was not observed in other polycrystalline multilayer MoS_2_ thin films [26, 29] or single crystals [9, 16, 30] after O_2_ plasma or UV-O_3_ treatment. While this inconsistency may be related to dose- and time-dependence of MoS_2_ oxidation [30], more systematic investigation is needed to clarify this in the future.

The different XPS behavior may be related to the difference of composition and crystallinity between single crystals and CVD thin films. The composition of Mo:S is 1:1.97 in bulk single crystals and 1:1.5 in CVD thin films, suggesting higher concentration of S vacancies in CVD thin films. The higher concentration of S vacancies, combined with the existence of grain boundaries in CVD thin films, may allow higher reactivity to oxygen than that in single crystals.

**Fig. 6:**
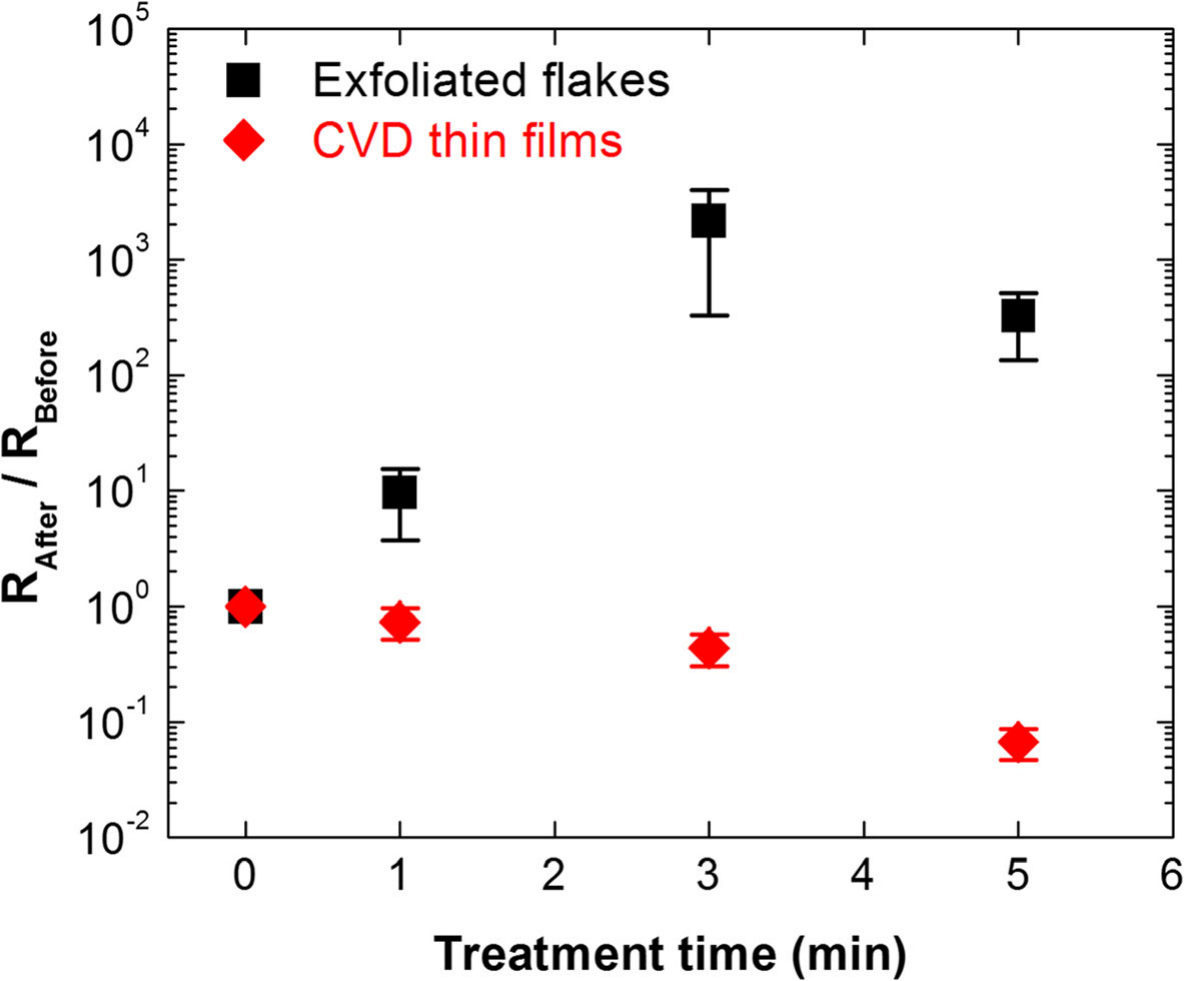
Ratio of electrical resistance of MoS_2_ monolayers as a function of UV-O_3_ treatment time (*R*_After_: electrical resistance after UV-O_3_ treatment, *R*_Before_: electrical resistance before UV-O_3_ treatment)

To further understand the oxidation of MoS_2_ monolayers by UV-O_3_ treatment, we measure the electrical resistance of pristine and UV-O_3_-treated MoS_2_ monolayers. Because there is sample-to-sample variation of electrical resistance, we use relative ratio of electrical resistance (*R*_After_/*R*_Before_), where *R*_After_ and *R*_Before_ are electrical resistance after and before UV-O_3_ treatment, respectively. Figure 6 shows *R*_After_/*R*_Before_ as a function of UV-O_3_ treatment time. While *R*_After_/*R*_Before_ of exfoliated MoS_2_ single crystal flakes significantly increases with longer treatment time, *R*_After_/*R*_Before_ of CVD MoS_2_ thin films decreases with longer treatment time. These results suggest that MoO_3_ formed by the UV-O_3_ treatment of CVD MoS_2_ thin films possesses higher doping level than that of MoS_2_ single crystal flakes. This is supported by XPS analysis suggesting the possible existence of oxygen vacancies, MoO_x_S_y_, or Mo (SO_4_)_x_ in CVD MoS_2_ monolayers. This is seemingly contradicting with the higher doping in single crystal MoS_2_ suggested in Fig. 5a. However, as Fig. 5a is based on bulk single crystals, we cannot exclude the possibility that it does not provide accurate information of the top monolayer. Hence, surface oxidation of bulk MoS_2_ single crystal may possibly provide doping only to MoS_2_ single crystal underneath, transforming top surface region into negligibly doped MoO_3_. Consistent with these results, electrical resistance also increased when monolayer MoS_2_ single crystal flakes were oxidized by O_2_ plasma [12]. As single crystal MoS_2_ without grain boundaries could be more tolerant to oxidation than polycrystalline MoS_2_, the effect of oxidation-induced doping may be stronger in polycrystalline MoS_2_ than in single crystal MoS_2_. However, further investigation is needed to understand this difference in the future.

## Conclusions

In summary, we investigated the effect of UV-O_3_ treatment on polycrystalline CVD thin films and single crystal flakes of monolayer MoS_2_. Monolayer MoS_2_ becomes transparent after UV-O_3_ treatment suggesting the formation of wide bandgap semiconductor MoO_3_. As UV-O_3_ treatment time increases, the intensity of PL and Raman spectra significantly decreased, suggesting the formation of oxides or defects. In both MoS_2_, XPS analysis indicated the formation of Mo-O bonds and MoO_3_. However, in CVD MoS_2_ thin films, the conversion of Mo^4+^-and S^2−^-states into Mo^5+^- and S^6+^-states was also observed after UV-O_3_ treatment, suggesting the possible existence of oxygen vacancies, MoO_x_S_y_, or Mo (SO_4_)_x_. As the electrical resistance of single crystal MoS_2_ monolayers significantly increased with longer UV-O_3_ treatment time, the oxidation of single crystal MoS_2_ into MoO_3_ seems to provide negligible doping. In contrast, the electrical resistance of CVD MoS_2_ monolayers decreased with longer UV-O_3_ treatment time, suggesting that the oxidation of CVD MoS_2_ into MoO_3_ provides doping. These results demonstrate the significant impact of crystallinity on the effect of UV-O_3_ treatment on MoS_2_ monolayers, providing possibly interesting implications on fabricating heterojunction structures based on two-dimensional nanomaterials.

## Additional File

Additional file 1: Crystallinity and AFM analysis. (DOCX 13198 kb)

## Data Availability

All data and materials may be provided on a reasonable request.

## Abbreviations

CVD: Chemical vapor deposition
I-V: Current-voltage
R_After_: Resistance measured after ultraviolet-ozone treatment
R_Before_: Resistance measured before ultraviolet-ozone treatment
TMDs: Transition metal dichalcogenides
UV-O_3_: Ultraviolet-ozone
XPS: X-ray photoelectron spectroscopy
