# Supplementary material for: Effect of Ultraviolet-Ozone Treatment on MoS2 Monolayers: Comparison of Chemical-Vapor-Deposited Polycrystalline Thin Films and Mechanically Exfoliated Single Crystal Flakes
Source: Nanoscale Res Lett. 2019 Aug 15;14:278. doi: 10.1186/s11671-019-3119-3 (PMC6695460; doi:10.1186/s11671-019-3119-3)
Supplement: Supplementary file 1 — Crystallinity and AFM analysis. (DOCX 13198 kb) [file 11671_2019_3119_MOESM1_ESM.docx]

Additional file 1

Effect of ultraviolet-ozone treatment on MoS_2_ monolayers: comparison of chemical-vapor-deposited polycrystalline thin films and mechanically-exfoliated single crystal flakes

Changki Jung, Hae In Yang, and Woong Choi

School of Materials Science & Engineering, Kookmin University, Seoul 02707, Korea

**1. Crystallinity of bulk MoS_2_ crystals and monolayer MoS_2_ thin films**

XRD and TEM analysis indicated the single crystal nature of bulk MoS_2_ crystals and polycrystalline nature of monolayer MoS_2_ thin films, respectively.

Figure S1. (a) XRD pattern of a bulk MoS_2_ crystal with crystallographic family of (00*l*) reflections indicating its single crystal nature and (b) TEM selected area electron diffraction (SAED) pattern with rings indicating polycrystalline nature of monolayer MoS_2_ thin films.

**2. AFM analysis**

AFM analysis indicated an increase of root-mean-squared (RMS) surface roughness after 5-minute UV-O_3_ treatment. The RMS roughness increased from 0.701 nm to 2.627 nm for a mechanically-exfoliated monolayer MoS_2_ flake and from 0.564 nm to 1.384 nm for CVD MoS_2_ thin films.

Figure S2. AFM images (5 μm × 5 μm) of an exfoliated MoS_2_ flake (a) before and (b) after 5-minute UV-O_3_ treatment and CVD MoS_2_ thin films (c) before and (d) after 5-minute UV-O_3_ treatment.
